# Intestinal barrier compromise, viral persistence, and immune dysregulation converge on neurological sequelae in Long COVID

**DOI:** 10.3389/fnagi.2025.1744415

**Published:** 2026-01-20

**Authors:** Laurence Leclerc, Johanne Poudrier, Christopher Power, Grace Y. Lam, Emilia Liana Falcone

**Affiliations:** 1 Center for Immunity, Inflammation and Infectious Diseases, Montreal Clinical Research Institute (IRCM), Montreal, QC, Canada; 2 Department of Microbiology, Infectious Diseases and Immunology, Université de Montréal, Montreal, QC, Canada; 3 Division of Neurology, Department of Medicine, Faculty of Medicine & Dentistry, University of Alberta, Edmonton, AB, Canada; 4 Department of Medicine, Faculty of Medicine & Dentistry, University of Alberta, Edmonton, AB, Canada; 5 Division of Pulmonary Medicine, Department of Medicine, University of Alberta and Alberta Health Services, Edmonton, AB, Canada; 6 Alberta Respiratory Centre, University of Alberta, Edmonton, AB, Canada; 7 Women and Children's Health Research Institute, University of Alberta, Edmonton, AB, Canada; 8 Department of Medicine, Université de Montréal, Montreal, QC, Canada; 9 Division of Microbiology and Infectious Diseases, Department of Medicine, Centre Hospitalier de l'Université de Montréal (CHUM), Montreal, QC, Canada

**Keywords:** gut dysbiosis, intestinal barrier dysfunction, Long COVID, microbial translocation, myeloid activation, neuroinflammation, SARS-CoV-2, viral persistence

## Abstract

Long COVID (LC) is a multisystem, post-infectious conditions diagnosed ≥3 months after acute SARS-CoV-2 infection and marked by relapsing, persistent, or progressive symptoms, especially fatigue, post-exertional symptom exacerbation and neuropsychiatric syndromes. We synthesized evidence suggesting that LC arises from intersecting pathways including viral persistence, intestinal dysbiosis and barrier compromise with microbial translocation, innate immune activation with neutrophil extracellular traps (NET) and thromboinflammation, and immune dysregulation with features of exhaustion and autoimmunity. These processes adversely impact blood-brain barrier (BBB) function and lead to neuroinflammation. We propose a mechanistic model in which viral antigens and translocated microbial products amplify pro-inflammatory networks promoting immunothrombosis and tissue hypoperfusion. Hematogenous and gut-brain pathways may then deliver inflammatory mediators to the central nervous system (CNS), resulting in BBB disruption and glial activation that underpin nervous system disorders in LC. Treatment regimens aimed at lowering antigen load, restoring mucosal barrier integrity and modulating myeloid/coagulation pathways may warrant investigation as novel therapeutic strategies to treat LC.

## 1 Introduction

### 1.1 SARS-CoV-2 infection

Severe acute respiratory syndrome coronavirus 2 (SARS-CoV-2), the causative agent of coronavirus disease 2019 (COVID-19), is primarily transmitted through aerosols that enter the body via the respiratory tract and initially target the airway epithelium. As the viral entry process has been reviewed extensively (Jackson et al., 2022), we summarize only the key steps here. The viral spike (S) glycoprotein comprises two subunits: S1, which binds the angiotensin-converting enzyme 2 (ACE2) receptor (highly expressed on alveolar type II cells), and S2, which mediates viral fusion after cleavage by host transmembrane protease serine 2 (TMPRSS2). Following receptor engagement and protease-dependent activation, virions enter cells (via fusion at the plasma membrane or endocytosis), release their positive-sense RNA genome and initiate replication and translation of viral proteins (Lamers and Haagmans, 2022). Newly assembled virions are then released from the infected cells by exocytosis (Ghosh et al., 2020). In addition to ACE2, SARS-CoV-2 can interact with other pattern recognition receptors (PRR) such as C-type lectins e.g., dendritic cell-specific intercellular adhesion molecule 3 (ICAM-3)-grabbing non-integrin (DC-SIGN) and liver/lymph node-specific ICAM-3-grabbing non-integrin (L-SIGN) that may facilitate viral capture and trans-infection by dendritic cells without replication (Lu et al., 2021; Thépaut et al., 2021), similar to mechanisms described in human immunodeficiency virus (HIV; Hodges et al., 2007).

**Figure 1:**
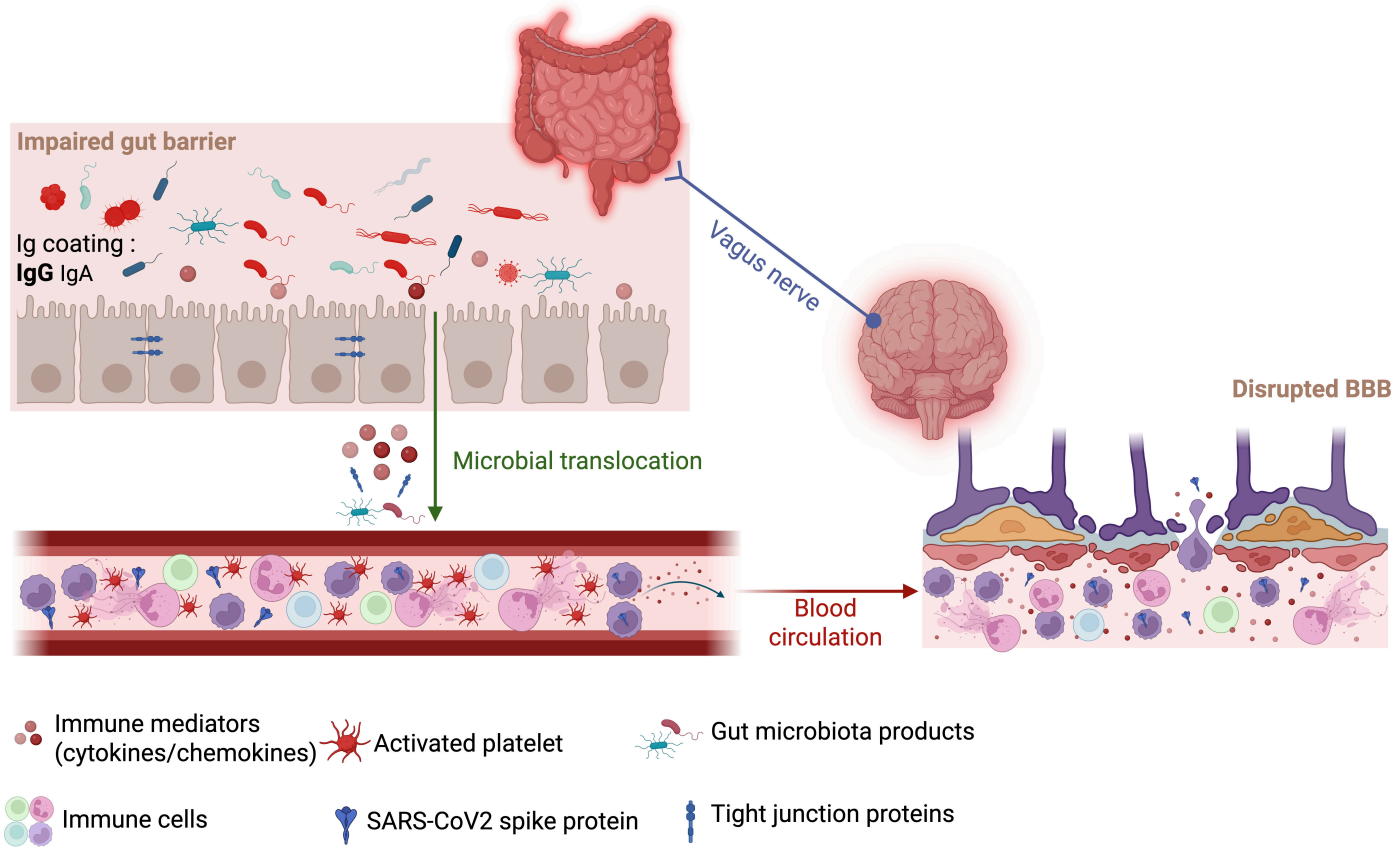
Proposed gut-brain axis linking intestinal barrier dysfunction to neuroinflammation in Long COVID. Conceptual schematic of how viral persistence and systemic inflammation may converge on the gut-brain axis in Long COVID (LC; Created in BioRender. Massé, C. (2026) https://BioRender.com/xmhwcwi). **Left:** In the intestine, inflammation, dysbiosis and epithelial tight-junction disruption weaken the barrier and alter IgA/IgG coating of microbes. This promotes microbial translocation (e.g., lipopolysaccharide [LPS], LPS-binding protein [LBP], β-D-glucan) and release of immune mediators. SARS-CoV2 spike antigen may also be present locally. **Center** (blood): Translocated microbial products and viral antigens sustain innate immune activation (myeloid cells, cytokines/chemokines), associate with platelet activation/thromboinflammation, and circulate to distal tissues. **Right** (brain): Circulating mediators and antigens contribute to blood-brain barrier (BBB) dysfunction with loosened tight junctions, permitting entry of inflammatory signals and cells and thereby amplifying neuroinflammation. A vagus-nerve pathway is shown as a parallel route for gut-to-brain signaling.

Although COVID-19 is classically a respiratory disease, ACE2 is expressed in many organs, including the pancreas, kidney, gastrointestinal (GI) tract and central nervous system (CNS) (Jackson et al., 2022). Consistent with this, many patients experience GI symptoms, such as diarrhea, nausea and vomiting during their acute infection (Groff et al., 2021). In fact, intestinal epithelial cells (enterocytes) exhibit among the highest ACE2 expression and co-express relevant proteases, supporting the possibility of direct intestinal infection (Assimakopoulos et al., 2022; Lamers et al., 2020). Viral involvement in the gut can promote local inflammation, intestinal dysbiosis, and impaired epithelial barrier integrity (i.e., “leaky gut”), which together can increase microbial translocation and alter immunoglobulin (Ig) coating of commensal bacteria. In the context of intestinal inflammation, studies have demonstrated that ulcerative colitis is associated with a shift in the relative balance of antibody-coated microbes, characterized by an increased proportion of IgG-coated bacteria and a reduced predominance of IgA-mediated coating. This altered IgA to IgG coating ratio reflects a breakdown in mucosal immune homeostasis and is associated with increased pro-inflammatory responses (Castro-Dopico et al., 2019). Similar disruptions in Ig coating have also been described in other inflammatory conditions affecting the gut, including chronic HIV infection (Buckner et al., 2013, 2014). Together, these events may contribute to systemic immune dysregulation and neuroimmune signaling that set the stage for long-term sequelae (Figure 1).

Typically, coordinated innate and adaptive immune responses clear viral progeny and infected cells. However, in a subset of individuals, especially those with severe COVID-19 infection and/or dysregulated immunity, viral clearance may be delayed or incomplete, thereby increasing the risk of post-infectious conditions, and in some cases, life-threatening complications. Epidemiological estimates suggest that a substantial proportion of individuals report persistent symptoms following even a mild COVID-19 infection (Hou et al., 2025). The high burden of post-COVID-19 condition (PCC or Long COVID) has shed light on other post-infectious complications, which together justify the need to further our understanding of the pathophysiological underpinnings of these conditions.

### 1.2 Long COVID definition

Long COVID (LC) is a post-infectious, multisystem chronic condition that follows acute SARS-CoV-2 infection, is usually diagnosed at least 3 months from the onset of infection, with symptoms lasting for at least 2 months that are not explained by an alternate diagnosis (Soriano et al., 2022; Ely et al., 2024). Although the clinical presentation can be heterogeneous (i.e., up to 200 symptoms have been reported; Davis et al., 2021) and include new, relapsing and/or persistent symptoms, the most common LC features include intense fatigue, post-exertional malaise/post-exertional symptom exacerbation (PEM/PESE) and cognitive dysfunction described as brain fog, manifesting as trouble with memory and/or concentration (Ely et al., 2024). In this Review, we focus on mechanisms that plausibly link viral persistence, intestinal barrier dysfunction, immune dysregulation and neuroinflammation to these clinical manifestations.

### 1.3 Risk factors predisposing to Long COVID

Acute SARS-CoV-2 infection ranges from asymptomatic to severe disease requiring hospitalization or resulting in death (Brodin, 2021). Severity profiles likely reflect a combination of host genetics, age/sex, comorbidities and baseline immune status. Defects in type I interferon (IFN) pathways including inborn errors of immunity (Zhang Q. et al., 2020) or neutralizing anti-IFN autoantibodies (Bastard et al., 2020) are strongly associated with severe, life-threatening COVID-19. LC can follow asymptomatic or mild/moderate acute SARS-CoV-2 infection, though higher prevalence is consistently reported among those hospitalized during their acute illness (Lu et al., 2024). Early viral kinetics and quality/timing of host immune responses likely shape downstream LC risk (Su et al., 2022). Notably, while anti-type I IFN autoantibodies are key mediators of acute disease severity, they do not appear to drive LC (Rodriguez et al., 2024). Similarly, high levels of circulating IL-6 do not appear to predispose patients to developing chronic fatigue syndrome following acute COVID-19 (Freidin et al., 2023). Additional risk factors associated with LC include SARS-CoV-2 RNAemia, type 2 diabetes, Epstein-Barr virus (EBV) viremia and earlier variants of SARS-CoV-2 (Su et al., 2022; Lok et al., 2024). A genome-wide association study identified *FOXP4* variants as being associated with increased LC risk (Lammi et al., 2025).

Across cohorts and meta-analyses, frequently reported risk factors for LC include pre-existing comorbidities, female sex, older age, high body mass index, and smoking (Tsampasian et al., 2023), but not the presence of inborn errors of immunity (Leeuwen et al., 2025). Phenotypically, LC comprises heterogeneous endotypes, with subsets sharing features such as viral persistence and coagulopathy, including microclot formation (Pretorius et al., 2021). Although vaccination reduces both the risk and severity of LC, it does not fully prevent its development (Al-Aly et al., 2022) nor consistently eliminate viral persistence when present (Nayyerabadi et al., 2023). In this context, early antiviral treatment, such as administration of nirmatrelvir/ritonavir (Paxlovid) during acute SARS-CoV-2 infection, has been associated with a reduced risk of developing post-acute sequalae and may represent a potential strategy for LC prevention (Xie et al., 2023).

## 2 Drivers of sustained inflammation in Long COVID

### 2.1 SARS-CoV-2 systemic infection

Systemic spread of SARS-CoV-2 is a plausible driver of sustained inflammatory tone observed in LC. ACE2, the viral entry receptor, is expressed on epithelial and endothelial cells across multiple organs, including the lung, vasculature, pancreas, and intestine (Hamming et al., 2004). Beyond serving as a viral receptor, ACE2 is a key component of the renin-angiotensin system, with roles in blood pressure regulation and local organ function (Perlot and Penninger, 2013). In intestinal epithelial cells, ACE2 promotes B^0^AT1/SLC6A19 surface expression, enabling tryptophan uptake (Hashimoto et al., 2012). Previous studies have reported ACE2 downregulation following SARS-CoV-2 infection (Lu et al., 2022); in enterocytes, this could reduce tryptophan availability, since surface expression of the B^0^AT1 receptor requires ACE2 co-expression. Tryptophan is normally metabolized by gut epithelial cells via the mTOR pathway and leads to the production of antimicrobial peptides (Banu et al., 2020). Thus, ACE2 downregulation can potentially lower levels of antimicrobial peptides thereby reshaping the composition of the gut microbiota (Penninger et al., 2021).

Altered tryptophan-serotonin metabolism has also been observed in viral infections. Specifically, reduced peripheral serotonin (5-HT) levels were reported in acute COVID-19 and in LC and attributed to impaired tryptophan absorption downstream of ACE2 changes in the intestinal epithelium (Wong et al., 2023). Lower 5-HT may impact vagus nerve signaling that affects cognition and mood, thereby offering a mechanistic explanation for certain neuropsychiatric symptoms seen in LC (Wong et al., 2023). Similar ACE2-related functional perturbations have been described in the pancreas, in which infection and/or ACE2 downregulation correlated with pancreatic injury, episodes of hyperglycemia, and new-onset diabetes in some patients (Wihandani et al., 2023).

Collectively, these findings suggest that extrapulmonary infection and tissue-specific ACE2 perturbations contribute to organ dysfunction, systemic inflammation, and post-infectious complications.

### 2.2 Viral persistence

The chronic inflammatory state in LC suggests that ongoing stimuli sustain activation of the immune system after the initial infection (Nayyerabadi et al., 2023). A leading hypothesis is that persistence of viral material (proteins and/or RNA) sustains immune activation (Al-Aly and Topol, 2024).

#### 2.2.1 Viral proteins (spike and nucleocapsid)

LC cohorts have measured circulating SARS-CoV-2 antigens, before the emergence of delta and omicron variants (full length spike, S1 spike subunit and nucleocapsid) in plasma over time (Swank et al., 2023, 2024). Overall, antigen detection varies based on time from the acute infection. Specifically, nucleocapsid detection is generally more proximal to infection, whereas spike/S1 detection has been reported in some LC cohorts months to 1 year post-infection (Schultheiß et al., 2023), bringing forward spike as a potential LC biomarker. Spike (especially from the wild-type SARS-CoV-2 strain) can amplify lipopolysaccharide (LPS)-triggered inflammation (Samsudin et al., 2022), and owing to its extensive glycosylation, can engage PRRs such as DC-SIGN, L-SIGN and other C-type lectins on innate immune cells (Thépaut et al., 2021). Reports have identified intracellular spike in non-classical (CD14^low^/CD16^+^) monocytes independent of the vaccination status (Nayyerabadi et al., 2023), and up to 15 months post-infection (Patterson et al., 2022). Since non-classical monocytes display low ACE2 compared to classical monocytes (Rutkowska-Zapała et al., 2015), uptake may occur via lectins, Fc receptors, micropinocytosis, or transfer during monocyte maturation (Patterson et al., 2022), rather than direct ACE2-mediated entry. Persistent intracellular spike and PRR engagement may result in chronic myeloid activation, which is consistent with elevated myeloid activation markers in LC (Schultheiß et al., 2023). Notably, SARS-CoV-2 antigens are not uniformly detectable in blood. In one study, serologic and cellular profiles better reflected LC status (Rodriguez et al., 2024). As such, more severe LC profiles (often after mild-moderate acute infections) are associated with higher spike-specific IgG and constrained memory CD8^+^ T-cell expansion, while lower spike-specific IgG levels with increased frequencies of spike-specific CD8^+^ T-cells correlated with a better prognosis (Rodriguez et al., 2024). Such immune signatures are compatible with ongoing antigen exposure in a subset of patients.

#### 2.2.2 Viral RNA

Multiple studies have identified SARS-CoV-2 RNA and/or proteins in pulmonary and extrapulmonary tissues months after clinical recovery and negative nasopharyngeal PCR tests (Proal et al., 2023), linking LC to tissue persistence, and in some reports, higher tissue viral burden (Zuo et al., 2024). Although replication competence of the persistent viral RNA is yet to be demonstrated, observation of double stranded RNA (dsRNA) in gut lamina propria with nearby macrophages/monocytes suggest ongoing replication or replication intermediates in immune-monitored niches (Peluso et al., 2024b). Other proposed reservoirs include megakaryocytes and platelets, which can harbor infectious virus in severe acute COVID-19 (Zhu et al., 2022) and have been hypothesized to contribute to LC (He et al., 2014). Additionally, a study found that reverse transcription (RT)-droplet digital PCR (ddPCR) could be used to detect very low viral load, as opposed to standard qPCR-testing, revealing an interesting tool that could be used to detect persistent viral RNA in LC (Morón-López et al., 2023).

The concept that antigen persistence in long-lived or immune privileged cells can sustain inflammation has already been described in the context of chronic HIV infection, where viral products (e.g., Nef) can be released from latently infected cells and captured by immune cells, thereby perpetuating activation (Ferdin et al., 2018; Chagnon-Choquet et al., 2015). Therefore, in the context of LC, tissue dsRNA plus circulating viral proteins support the presence of hidden reservoirs [theorized to include the central nervous system and GI tract (Peluso et al., 2024b; Stein et al., 2022)] that may continually release antigen, thereby maintain inflammation and potentially affect immune competence and quality of responses to vaccination. Persistent pathogen-derived materials driving inflammation is a theme shared with malaria (plasmodium pigment hemozoin; Olivier et al., 2014), HIV (gp120, nef; Chagnon-Choquet et al., 2015; Poudrier et al., 2001; Benlarbi et al., 2024) and SARS-CoV2 spike in LC.

### 2.3 Dysbiosis, leaky gut, and microbial translocation

SARS-CoV-2 involvement of the gut mucosa can establish a self-reinforcing cycle in which microbiome imbalance leads to epithelial barrier disruption, permitting microbial products to cross into systemic circulation and activate innate immune pathways that amplify systemic inflammation. Notably, spike proteins alone can induce pro-inflammatory profiles in human enterocytes (Nascimento et al., 2024). Consistent with this model, studies in LC cohorts have shown intestinal dysbiosis, evidence of epithelial tight-junction injury, and elevated plasma markers of microbial translocation (Giron et al., 2022; Liu et al., 2022). This would suggest that particles translocating into circulation could potentially interact with and cross over into end organs such as the CNS (Zeiher et al., 2025; further discussed in neuroinflammation section).

#### 2.3.1 Altered gut microbiota in Long COVID

Across studies, acute COVID-19 is associated with increases in opportunistic/pathobiont taxa and reduced beneficial commensals (Liu et al., 2022). Individuals who recover from COVID-19 typically revert toward a healthier microbial profile, whereas people with LC often show persistent, pro-inflammatory shifts at 6 months post-infection (Liu et al., 2022), including higher *Ruminococcus gnavus* and *Bacteroides vulgatus* and lower *Faecalibacterium prausnitzii*, together with a reduction in short-chain fatty-acid (SCFA) producing taxa and lower butyrate levels (Liu et al., 2022). Diminished SCFAs can weaken immunoregulatory crosstalk as SCFAs interact with membrane protein receptors on immune cells leading to inhibition of the NF-kB-mediated inflammatory programs (Zhang et al., 2023). Accordingly, microbiome dysbiosis and reduced SCFAs provide a plausible mechanism for the sustained inflammatory state observed in LC.

#### 2.3.2 Damage to epithelial cell tight junctions

The intestinal barrier depends on a single layer of epithelial cells sealed by tight junction complexes (e.g., claudins, occludins, ZO-1) that maintain selective permeability. In LC, the pro-inflammatory mucosal environment is associated with disruption of these complexes and impaired barrier function (Zhang et al., 2024). As mentioned above, SARS-CoV-2-associated dysbiosis and reduced SCFAs (notably butyrate) can reduce IL-22-driven epithelial/immune programs. In the gut, IL-22 helps sustain a balanced microbiota and strengthen the intestinal barrier by promoting mucus and antimicrobial peptide production (Jayasimhan and Mariño, 2019; Howell et al., 2021), thereby protecting the intestinal epithelium from pathogens (Keir et al., 2020). Lower IL-22 activity, together with direct epithelial injury from SARS-CoV-2 and pro-inflammatory cytokines [e.g., Tumor necrosis factor (TNF) or Interferon-gamma (IFN-g)] infection in gut leads to poorer intestinal barrier integrity.

Consistent with intestinal barrier compromise, people with LC show elevated zonulin, a tight junction protein, alongside plasma markers of microbial translocation such as LPS-binding protein (LBP) and β-glucan, reflecting bacterial and fungal products, respectively (Giron et al., 2022). Additionally, higher levels of soluble CD14 (sCD14) have been detected in LC cohorts, indicating monocyte activation in the context of LPS exposure (Rohrhofer et al., 2025). These microbial ligands engage PRRs [e.g., Toll-like receptors (TLRs)] on innate immune cells, thereby promoting inflammatory signaling.

Altogether, shifts in the microbiota changes and activation of local immune responses can compromise intestinal epithelium tight junction integrity. The resulting translocation of microbial products sustains chronic activation of innate immune cells and contributes to the systemic inflammatory burden described in both acute COVID-19 and LC, with downstream effects on immune cell composition and immune competence (Talwar et al., 2025).

## 3 Immune dysregulation

The chronic inflammatory state in LC indicates sustained immune activation beyond the acute phase. Whether driven by persistent stimuli (e.g., viral antigens/RNA, microbial products) and/or intrinsic dysregulation of immune circuits, accumulating evidence supports multilayered immune dysregulation in LC pathology.

### 3.1 Innate immunity (myeloid, epithelial, endothelial cells, neutrophils)

Plasma biomarker studies consistently show elevated pro-inflammatory cytokines/chemokines in LC. Higher levels of IL-6, IL-8, TNF-α, IL-1β, and CXCL10 during acute COVID-19 have been associate with post-infectious sequelae (Peluso et al., 2024a), and IL-6, TNF-α and IL-1β can remain elevated months later in LC cohorts (Wang et al., 2023). Additional reports describe increases in IL-5, IL-9, IL-17F, IL-22, IL-23, and IL-33 and persistent type I/III IFN signatures up to 8 months post-COVID-19 (Phetsouphanh et al., 2022). These mediators, often produced by myeloid, epithelial, and endothelial cells, suggest ongoing activation of first-line innate populations.

Cellular analyses show increased CD38^+^/HLA-DR^+^ myeloid cells and activated intermediate monocytes (CD14^+^/CD16^+^) up to 8 months post-infection (Phetsouphanh et al., 2022). Severe acute COVID-19 can imprint epigenetic/transcriptional programs in monocytes and hematopoietic stem/progenitor cells thereby increasing myelopoiesis and promoting pro-inflammatory, pro-migration phenotypes for up to a year post-infection (Cheong et al., 2023). Given the persistent pro-inflammatory cytokine/chemokine milieu in LC (Nayyerabadi et al., 2023), similar skewing may perpetuate myeloid-driven inflammation, creating a feed-forward loop of cytokine/chemokine release and bystander activation of other immune cells.

Neutrophil activation also appears to be sustained in LC and likely contributes to perpetuating inflammation. Proteomic profiling (Olink) has highlighted neutrophil degranulation pathways and increased matrix metalloproteinase 8 [an enzyme released during neutrophil extracellular trap (NET) formation] as correlating with increased blood neutrophil counts in LC (Woodruff et al., 2023). In patients with post-COVID-19 lung fibrosis, myeloperoxidase and citrullinated histone 3 (H3cit) remain elevated in plasma up to 6 months after infection (George et al., 2022), consistent with ongoing NET formation.

Innate activation appears to intersect with endothelial injury and coagulation in LC. Beyond direct effects of viral products on the endothelium (Jover et al., 2021), LC cohorts show elevated von Willebrand factor, tissue factor (TF), Factor VIII, and proteomic signatures of thromboinflammation and complement activation (Turner et al., 2023). Amyloid deposit/microclots resistant to fibrinolysis have also been reported in LC (Pretorius et al., 2021). Activated monocytes can express TF, bind platelets and interact with the endothelium, while NETs activate the endothelium and trap platelets, together promoting immunothrombosis and microvascular occlusion (Bonaventura et al., 2021). Emerging data implicate hypoxia inducible factor 1 alpha (HIF-1α) pathway dysregulation in LC (da Silva et al., 2025). Indeed, while HIF-1α can repress ACE2/TMPRSS2 in lung epithelium, it may have opposite effects in endothelium, supporting a vascular-proliferative phenotype linked to progression from acute COVID to LC (Iosef et al., 2023; Patel et al., 2022). Collectively, persistent innate activation, potentially maintained by viral antigens and/or translocated microbial products, may drive a pro-coagulant state that could be associated with microclotting, and tissue hypoxia, offering a unifying explanation for symptom heterogeneity in LC (Turner et al., 2023).

### 3.2 Adaptative immunity (T-cell, B-cell, and autoimmunity)

LC has been associated with features of lymphocyte dysfunction, from exhaustion markers to bystander/polyclonal activation, with potential erosion of immune competence resembling aspects of immunosenescence (Wallis and Williams, 2022). Dysregulated adaptive responses are reported in both severe acute COVID-19 infection (Moga et al., 2022) and LC (Klein et al., 2022).

Studies suggest that virus-specific memory can persist. Individuals with LC maintain SARS-CoV-2-specific T- and B-cell populations for many months, with CD4^+^ and CD8^+^ clonotypes detectable >2 years after infection (Rowntree et al., 2024). Another study, found minimal differences in virus-specific CD4+ and CD8+ T-cells compared to controls, although inhibitory receptors PD-1 and TIM-3 were modestly increased on SARS-CoV-2 non-spike-specific CD8^+^ T-cells in individuals with LC (Gao et al., 2025). Nevertheless, improper coordination between cellular and humoral arms of adaptive immunity has been described (Yin et al., 2024), consistent with reports of viral persistence in some individuals up to 24 months (Peluso et al., 2024b).

On the B-cell axis, LC and severe COVID-19 have been linked to dysregulated B-cell phenotypes, including T-bet^hi^/CD11c^+^ populations (often termed age-associated B-cells) that reflect extra-follicular responses, poor affinity maturation, and a propensity toward autoreactivity (Bastard et al., 2020; Knox et al., 2017; Nickerson et al., 2023; Kaneko et al., 2020; Zhang Y. et al., 2020; Wang et al., 2021; Chang et al., 2021), all of which suggesting contributions from first line B-cells (Doyon-Laliberté et al., 2022). Severe COVID-19 correlates with morbidity alongside these features (Hoehn et al., 2021). Post-mortem gastrointestinal (GI) studies of patients who died from COVID-19 show disrupted lymphoid architecture in ileal Peyer's patches with germinal center depletion and altered B- and T-cell zones (Trevelin et al., 2022). Early auto-reactivity has been reported as a risk factor for LC at the time of initial COVID-19 diagnosis (Su et al., 2022). Consistently, some LC cohorts exhibit lower SARS-CoV-2-neutralizing titers (Gao et al., 2025) and evidence of autoreactivity (Woodruff et al., 2023), with inverse relationships between autoantibody levels and virus-specific antibodies in some studies. Autoantibodies in LC may arise from tissue damage, molecular mimicry, or epitope spreading. To date, definitive LC- autoantibody signatures remain under investigation (Bodansky et al., 2023). Proteomic screens have identified markers of inflammation, altered B-cell responses, and autoreactivity >1 year post-infection (Woodruff et al., 2023). Links between NETs and autoantigen exposure further connect neutrophil activity to autoimmunity (Monsalve et al., 2025).

These observations echo patterns in systemic rheumatic disorders (e.g., lupus; Jenks et al., 2019) and chronic viral inflammation (e.g., HIV; Doyon-Laliberté et al., 2022), where dysregulated B-cell populations (sharing similarities with the populations described in the context of SARS-CoV-2) and interferon-skewed epigenetic profiles are likely to accumulate. Notably, “memory-like” B-cells generated in chronic lymphocytic choriomeningitis virus settings carry IFN stimulated genes (ISG)-enriched epigenetic signatures, illustrating how persistent inflammatory cues can imprint long-lived adaptive populations, a concept which is relevant to LC (Cooper et al., 2024).

## 4 How does immune dysregulation contribute to neuroinflammation

### 4.1 Evidence of neuroinflammation

LC carries a substantial neurological burden, including cognitive dysfunction (“brain fog”), headache, sleep disturbances, neuropathic pain/dysfunction and neuropsychiatric symptoms commonly manifesting with depression/anxiety (Greene et al., 2024). Depression and anxiety can be a LC symptom and/or a consequence of LC, which can sometime obscure the diagnosis of cognitive dysfunction. Imaging studies report reduced gray-matter thickness (Xu et al., 2022), overall brain volume loss (Douaud et al., 2022), and neurovascular abnormalities, with involvement of both central and autonomic nervous systems (Douaud et al., 2022). Common autonomic manifestations include postural orthostatic tachycardia syndrome (POTS), characterized by vasomotor dysregulation and reduced cardiac preload with compensatory tachycardia, and inappropriate tachycardia syndrome (Raj et al., 2021). Typical symptoms associated with POTS include dizziness, cognitive dysfunction, and fatigue (Arnold et al., 2018).

Cognitive dysfunction can follow both severe and mild acute SARS-CoV-2 infection (Becker et al., 2021). In a mouse model of mild SARS-CoV-2 respiratory infection, reactive microglia and pro-inflammatory cytokines/chemokines persisted in the brain and cerebrospinal fluid (CSF) 7 weeks post-infection, accompanied by reduced hippocampal neurogenesis and loss of myelinating oligodendrocytes (Fernández-Castañeda et al., 2022). In contrast, mild H1N1 infection induced partially overlapping hippocampal changes but did not cause a sustained oligodendroglial deficit, suggesting a SARS-CoV2-specific effect. In humans, however, clinical evidence of demyelination in LC remains limited and may be under-detected with current neuroimaging techniques (Khodanovich et al., 2022). Large LC cohort studies have not demonstrated an increased incidence of multiple sclerosis (MS) to date (Monje and Iwasaki, 2022), although longer-term follow-up may be warranted. Overall, demyelination driven by inflammation and/or autoreactivity remains a plausible but unconfirmed contributor to neurological symptoms in LC, which clinically overlap with MS, a prototypical demyelinating disease (Delgado-Alonso et al., 2024).

SARS-CoV-2 RNA and proteins have been detected in brain tissue at autopsy up to 7 months after symptom onset, generally with minimal direct viral cytopathology outside the respiratory tract (Stein et al., 2022). This supports a model in which replication competence in brain of an animal model of LC has been demonstrated up to 21 days post-infection with associated increased pro-inflammatory marker, IL-6, and depression/anxiety-like behaviors (Ogando et al., 2025). Hypoxia also appears to be contributory, as similar amyloid-like deposits were found in the brains of individuals who died from COVID-19 as compared to individuals who died from hypoxia or ischemia (Priemer et al., 2022). In LC, [11C]-PBR28 PET neuroimaging has linked neuroinflammation with circulating markers of vascular dysfunction (VanElzakker et al., 2024), and decreased neurovascular perfusion correlates with persistent cognitive complaints (Ajčević et al., 2023). Autopsy studies have revealed microvascular injury including fibrinogen leakage, endothelial basement membrane thinning in the olfactory bulb (Lee et al., 2021), serum protein extravasation, platelet accumulation, and activation of the coagulation system (Lee et al., 2022).

Together, these findings support a model in which systemic inflammation, microclotting-associated hypoxia, and blood-brain barrier (BBB) perturbation drive CNS immune activation in LC, even though overt viral replication in the brain appears to be infrequent (Monje and Iwasaki, 2022).

### 4.2 Blood-Brain-Barrier dysfunction in Long COVID

The BBB is formed by specialized endothelial cells connected by tight junctions and supported by pericytes, a basement membrane, and astrocytic end-feet, and it tightly regulates molecular and cellular trafficking into the CNS (Dotiwala et al., 2025). Several tight junction-associated proteins expressed at the BBB, including occludin and claudins, are also present in the intestinal epithelial barrier. However, their organization and functional stringency differ substantially. The BBB is an exceptionally selective barrier that is normally shielded from direct exposure to microbial and viral antigens, whereas the intestinal barrier is continuously exposed to microbial products and immune cells (Daneman and Rescigno, 2009). In the context of LC, a sustained pro-inflammatory milieu may disrupt BBB integrity by impairing endothelial function and junctional organization. Pro-inflammatory cytokines such as IL-6, IL-1β, and TNF-α, as well as MMPs, have been shown to compromise tight junctions through altered junctional complex organization and increased transcellular permeability (Huang et al., 2021; Versele et al., 2022). Pericytes express relatively high levels of ACE2, rendering them potentially susceptible to SARS-CoV-2 infection either via systemic inflammation and/or infiltration of infected leucocytes into the CNS. Experimental data suggest that such infection can promote pericyte constriction, detachment, and/or loss (Khan et al., 2022; McQuaid and Montagne, 2022), thereby disturbing their crosstalk with endothelial cells and likely contributing to vascular instability. The resulting barrier dysfunction may facilitate entry of inflammatory mediators (e.g., cytokines/chemokines), microbial products derived from gut translocation, circulating viral antigens, activated immune cells (myeloid cells, T and B cells), and autoantibodies, all of which can amplify CNS inflammation (Huang et al., 2021).

Downstream, microglia and astrocytes can become activated and release additional cytokines/chemokines and reactive oxygen species, thereby propagating neuroinflammation (Greene et al., 2024). Individuals with mild-moderate acute SARS-CoV-2 infection who develop LC show persistent depressive and cognitive symptoms associated with gliosis (Braga et al., 2023). Biomarkers of neuroinflammation/neuroaxonal injury such as glial fibrillary acidic protein, tau, and neurofilament light chain have been shown to be elevated in CSF in some LC cohorts (Rong et al., 2024) and can be increased in blood among patients presenting neurocognitive symptoms (Gutman et al., 2024), although blood levels may not always reliably indicate CNS injury (Comeau et al., 2023). Fibrin, a key blood clot component derived from fibrinogen, has emerged as a predictor of LC-related cognitive deficits (Taquet et al., 2023); it is deposited at sites of vascular damage/BBB disruption and can directly trigger innate immune responses in neurodegenerative diseases (Ryu et al., 2018). Notably, fibrin binds the SARS-CoV-2 spike protein and potentiates neuropathology (Ryu et al., 2024), and fibrin-spike presence in the brain can heighten microglia activation even without systemic infection.

Experimental data further show that spike protein administered intravenously can cross the murine BBB, likely via adsorptive transcytosis, and reach the brain parenchyma (Rhea et al., 2021). In human samples, spike accumulation has been reported along the skull-meninges-brain axis long after respiratory clearance and has been associated with neurodegenerative signatures. In mice, spike has the potential to induce neuroinflammation, proteomic remodeling in the skull-meninges-brain axis, anxiety-like behavior, and worsens outcomes after stroke/traumatic brain injury (Rong et al., 2024). Intracerebral spike infusion triggers neuroinflammation and long-term cognitive deficits in mice through TLR4-dependent pathways (Fontes-Dantas et al., 2023). These findings support the concept that persistent spike antigen, even without productive CNS infection, can drive neuroimmune pathology. However, in the context of LC, it is very unlikely that the spike protein would be crossing into the CNS at a high enough concentration to induce damage alone. As we have previously described, LC is a multi-factorial inflammatory condition that results in neurological pathologies caused by the combination of circulating pro-inflammatory cytokines, microbial/viral products and activated immune cells, rather than one specific pro-inflammatory molecule.

### 4.3 Gut-Brain axis in LC

The intestinal mucosa is densely innervated and communicates bidirectionally with the CNS via the gut-brain axis (Gareau and Barrett, 2023). Dysbiosis, intestinal barrier injury, and microbial translocation following SARS-CoV-2 infection can signal to the brain through vagal pathways and immune-neuroendocrine circuits, thereby modulating CNS function (Sittipo et al., 2022). The vagus nerve does not physically transport neurotransmitters from the gut to the brain but conveys neural signals shaped in part by gut-derived mediators, including serotonin, whose production is strongly influenced by the intestinal epithelium and microbiota (Wong et al., 2023). Under inflammatory conditions, changes in gut microbial composition can alter epithelial neurotransmitter production and other metabolites, emphasizing the importance of gut homeostasis for neuronal function. Additionally, dysbiosis-associated mucosal barrier impairment can facilitate translocation of microbial products and pro-inflammatory cytokines into the circulation. These mediators can act on the BBB, and when barrier integrity is compromised, access the CNS and promote neuroinflammation (Greene et al., 2024). Thus, mucosal inflammation and barrier dysfunction may contribute to neuroinflammatory and neurocognitive manifestations in LC. Supporting this concept, a recent study transplanted fecal microbiota from individuals with LC into germ free mice and observed impairments in memory, cognition, and spatial learning (Mendes de Almeida et al., 2023). These findings suggest that alterations in the intestinal microbiota can influence neurological function and may represent one pathway linking intestinal dysbiosis with neurological symptoms in LC.

## 5 Markers of Long COVID shared with other neuroinflammatory conditions: finding parallels to improve understanding of chronic immune dysregulation

Several viruses with neurotropic potential (e.g., measles virus, herpes virus, HIV) can cause CNS pathology. Although LC shares clinical and biological features with these conditions (summarized in Table 1), the neurological pathogenesis and long-term outcomes after SARS-CoV-2 infection require further clarification. Notably, post-infectious syndromes following respiratory viruses including respiratory syncytial virus, influenza, and human metapneumovirus, can also manifest with neurological complications (Bohmwald et al., 2018).

**Table 1:** Shared features among post-infectious conditions.

**Feature**	**Long COVID**	**Chronic HIV infection**	**Post-Ebola condition**	**Post-Lyme disease condition**	**ME/CFS**
Neurological symptoms	+	+	+	+	+
Evidence of pathogen persistence	+	+	+	+	
Evidence of neuroinflammation	+	+		+	
Evidence of autoimmunity	+	+		+	
Chronic innate immune activation	+	+	+		+
Evidence of intestinal dysbiosis	+	+			
Reactivation of other viruses (EBV, CMV, etc.)	+	+			+

HIV, human immunodeficiency virus; ME/CFS, myalgic encephalomyelitis/chronic fatigue syndrome; EBV, Epstein-Barr virus; CMV, cytomegalovirus.

### 5.1 Chronic neurological HIV condition

Amongst post-acute infectious chronic inflammatory states, HIV is the best characterized. Despite effective antiretroviral therapy, residual inflammation persists, associates with accelerated vascular aging (Aranguren et al., 2022) and cognitive impairment (Wallis and Williams, 2022), and can progress to HIV-associated neurocognitive disorders (Neuro-AIDS; Minagar et al., 2008). Ongoing morbidity is thought to reflect viral reservoirs that are not eradicated by anti-retroviral therapy (Mohammadzadeh et al., 2021) with ensuing viral protein release in the absence of high-level viral replication, thereby sustaining immune activation (Mohammadzadeh et al., 2023).

As in LC, BBB dysfunction with cytokine/chemokine signaling, leukocyte trafficking, and activation of astrocytes/microglia contributes to neuroinflammation in chronic HIV (Wallis and Williams, 2022). Molecular mimicry and autoreactivity may further amplify injury. SARS-CoV-2 proteins have sequence/structure homology with several self-proteins, including CNS antigens (Franke et al., 2023), some overlapping with MS-associated targets (Lake and Breen, 2023), supporting a plausible path to cross-reactivity.

Inflammation markers that indicate myeloid activation [e.g., IL-6, soluble CD163 (sCD163), sCD14] are relevant in HIV-related neurological disease (Wallis and Williams, 2022) and are elevated in COVID-19/LC in plasma and CSF. In severe COVID-19, sCD163, ferritin, and IL-18 associate with worse outcomes (Volfovitch et al., 2022; Marocco et al., 2022) and with neurological symptoms (Zingaropoli et al., 2023). sCD163 is also linked to HIV-associated neurocognitive impairment (Burdo et al., 2013). Shifts toward intermediate/non-classical monocytes occur in HIV (Wallis and Williams, 2022; Veenstra et al., 2019) and have been reported in LC, suggesting a shared myeloid signature. As in LC, intestinal dysbiosis and microbial translocation contribute to sustaining the inflammatory tone in chronic HIV (Parodi and de Rosbo, 2021; Monaco et al., 2016).

Both infections may also facilitate herpes viruses reactivation within the host virome (Monaco et al., 2016; Davis et al., 2023). Epstein-Barr virus (EBV) and cytomegalovirus (CMV) reactivation have been reported after COVID-19, with EBV more consistently linked to LC and neurological features (Gold et al., 2021; Peluso et al., 2022). EBV is a well-supported risk factor for MS in genetically/immune-primed hosts (Soldan and Lieberman, 2023) and is associated with several autoimmune diseases (Harley et al., 2018). Varicella-zoster virus (VZV) reactivation also appears to be increased post-COVID and in LC (Monje and Iwasaki, 2022), and in individuals with comorbidities, has been associated with rheumatic and cardiorenal complications (Lu et al., 2025; Chien et al., 2025). Herpes simplex viruses (HSV) reactivation causing severe complications (e.g., encephalitis) has been reported post-acute COVID-19 but remains uncommon in LC (Monje and Iwasaki, 2022; Gupta et al., 2022).

### 5.2 Post-Ebola condition

Post-Ebola syndromes (PES) share features with LC, including neurological symptoms and evidence of viral persistence (Caviness et al., 2017) in immune-privileged sites with intermittent viral RNA shedding for several months (up to 40 months in some reports; PREVAIL III Study Group et al., 2019; Deen et al., 2017; Mate et al., 2015). While some studies report limited association with classic inflammation markers (Tozay et al., 2020), other describe signatures enriched for PRR, IFN, and complement pathways, alongside immune dysregulation (Wiedemann et al., 2020). Recent data implicate monocyte/macrophage activation VEGF-A-mediated angiogenic signaling in PES (Wiedemann et al., 2020). A systematic review supports persistent inflammation and immune dysregulation in PES and in Lassa fever survivors, though evidence is heterogenous and sparse, limiting firm attribution of causality (Ficenec et al., 2025).

### 5.3 Post-Lyme disease condition

Post-treatment Lyme disease syndrome (PTLDS) can persist ≥6 months after *Borrelia burgdorferi* infection, with autonomic dysfunction being among prominent neurological complaints (Adler et al., 2024). Neuroimaging has demonstrated microglial activation in some PTLDS cohorts (Coughlin et al., 2018). While pathogen persistence remains debated (Adler et al., 2024), murine models demonstrate CNS colonization and inflammation by *B. burgdorferi*. It is thought that after treatment, non-viable bacterial fragments may still sustain neuroinflammation (Parthasarathy et al., 2013). Autoantibodies against neural antigens have been reported, which is consistent with molecular mimicry (Chandra et al., 2010).

### 5.4 Myalgic encephalomyelitis/chronic fatigue syndrome (ME/CFS)

ME/CFS is a chronic, often post-infectious condition characterized by symptoms that overlap with those reported in LC, including post-exertional malaise/post-exertional symptom exacerbation, non-restorative sleep, cognitive dysfunction, and dysautonomia. Although no single causative pathogen has been definitively identified, several viruses, including EBV, enteroviruses, herpesviruses, and parvovirus B19, have been proposed as potential triggers of ME/CFS. Recent epidemiological studies indicate that SARS-CoV-2 infection increases the risk of subsequently developing ME/CFS up to 4 years following acute infection. Nevertheless, LC and ME/CFS remain distinct clinical entities as LC is defined by its direct temporal association with SARS-CoV-2 infection, whereas ME/CFS can arise following a variety of infections (Hadidchi et al., 2025; Komaroff and Dantzer, 2025). Both conditions are associated with features of neuroimmune dysregulation, which may contribute to shared symptomatology and overlapping pathophysiological mechanisms. However, validated disease-specific biomarkers remain under investigation. Recent studies include a machine-learning classifier based on a panel of 11 micro-RNAs that discriminated ME/CFS from fibromyalgia in a research setting (Nepotchatykh et al., 2023), and increased circulating soluble SMPDL3 in ME/CFS, which is potentially related to monocytic activation (Rostami-Afshari et al., 2025). Additional work is exploring neurotransmitter receptor and ion-channel dysregulation as potential mechanistic contributors (Clarke et al., 2025). These candidate markers and pathways warrant systematic evaluation in LC given certain clinical and biological similarities with ME/CFS.

Across post-acute conditions, shared themes are emerging such as persistence of pathogen-derived antigens, innate-skewed systemic inflammation, intestinal dysbiosis and barrier leakage, loss of tolerance and dysregulated antibody responses (including potential cross-reactivity to neural antigens), BBB compromise, and microvascular as well as central and autonomic nervous system injury. While LC and HIV benefit from relatively robust research pipelines, several other post-infectious conditions remain under-investigated and represent a critical knowledge gap. Delineating overlaps in clinical phenotypes, mechanisms and biomarkers across these conditions may accelerate therapeutic development.

## 6 Conclusion

LC encompasses diverse endotypes and disease trajectories, implying multiple, intersecting mechanisms. Evidence points to roles for viral persistence, intestinal dysbiosis and barrier compromise, innate/myeloid activation with coagulopathy, adaptive immune dysregulation and autoreactivity, and neurovascular/BBB injury. Targeted functional studies are needed to define causal pathways, refine endotype-specific biomarkers, and guide precision therapy.

A practical implication, echoing lessons from HIV, is the value of early intervention. Where feasible, timely antiviral therapy during acute infection (Proal et al., 2025; Moir et al., 2010; Cai et al., 2024) may reduce inflammatory injury and risk of chronic sequelae. For individuals with LC, rational therapeutic combinations that address reservoirs/antigen load, restore intestinal barrier integrity, and modulate dysregulated immune/coagulation pathways may be required. Systematic comparisons across post-infectious syndromes can help identify shared targets and accelerate therapeutic development.
